# Ocular Inflammation and Infection

**DOI:** 10.1155/2012/403520

**Published:** 2012-10-04

**Authors:** Michelle Callegan, Meredith Gregory-Ksander, Mark Willcox, Susan Lightman

**Affiliations:** ^1^Department of Ophthalmology, University of Oklahoma Health Sciences Center and Dean McGee Eye Institute, Oklahoma City, OK 73104, USA; ^2^Schepens Eye Research Institute and Massachusetts Eye and Ear Infirmary, Harvard Mechanical school, Boston, MA 02114, USA; ^3^School of Optometry and Vision Science, University of New South Wales, Sydney, NSW 2052, Australia; ^4^Moorfields Eye Hospital NHS Foundation Trust, London EC 1V 2PD, UK

The eye is a unique and necessary organ that is constantly exposed to the environment. In an immune-privileged environment such as the eye, a delicate balance exists between immune responses that limit damage and those responses that can result in irreversible damage. Ocular inflammation and infection are therefore potentially blinding events. Efforts to analyze the delicate balance between helpful and harmful immune responses in the eye have involved a variety of animal studies and models which analyze not only the specific etiologic agents of infection and the contributions of their products in inflammation and vision loss but also the underlying host factors responsible for those responses. Other studies have probed the development of novel therapeutics based on endogenous host factors as well as pathogen-specific targets and have tested these with routinely used therapeutics in improved regimens in an effort to improve visual outcome. The ultimate goal is to provide patients with the appropriate treatment that rescues vision regardless of disease.

The scope of this special issue involves a wide variety of areas in ocular infection and inflammation highlighting recent developments in the epidemiology, pathogenesis, and treatment of various types of ocular infections and inflammation. The first paper of this special issue reviews the challenges of cataract surgery and visual rehabilitation in patients with uveitis. This discussion also includes management pearls in combating complications that arise in this particular group of patients. The second and third papers continue the theme of inflammation, analyzing chemotactic cytokine production in the cornea. The second paper reports a time course effect in the synthesis of IL-8 and MCP by stromal cells stimulated with LPS. The third paper uses an *ex vivo *model of herpes simplex virus 1 (HSV1) corneal infection to demonstrate that neutrophils are important in T-cell recruitment and control of HSV1 replication via synthesis of IP-10. The following three papers address epidemiological issues with studies on microbiological profiles and treatment outcomes of scleritis, chronic postoperative endophthalmitis, and fungal ocular infections. The key message of these studies is the importance of early identification and determination of drug susceptibility and proper therapeutic and/or surgical intervention in saving useful vision. The seventh and eight papers discuss the use of corticosteroid therapy in endophthalmitis and other types of ocular inflammation. The seventh paper reviews the visual outcome of cases of filtering bleb-associated endophthalmitis treated with or without intravitreal dexamethasone, while the eighth paper reviews the clinical use of loteprednol etabonate in a variety of ocular inflammatory conditions. The final paper of the special issue discusses hyperactivation of the renin-angiotensin system in inflammation and retinal neural dysfunction. The authors suggest that inhibition of this system may be a novel therapeutic approach to preventing or treating inflammation-based ocular diseases.

This special issue includes the following papers: “Cataract surgery in uveitis,” “IL-8 and MCP gene expression and production by LPS-stimulated human corneal stromal cells,” “Resident corneal cells communicate with neutrophils leading to the production of IP-10 during the primary inflammatory response to HSV-1 infection,” “Clinico-microbiological profile and treatment outcome of infectious scleritis: experience from a tertiary eye care center of India,” “Chronic postoperative endophthalmitis: a review of clinical characteristics, microbiology, treatment strategies, and outcomes,” “Support of the laboratory in the diagnosis of fungal ocular infections,” “Intravitreal dexamethasone in the management of delayed-onset bleb-associated endophthalmitis,” “Advances in corticosteroid therapy for ocular inflammation: loteprednol etabonate,” and “Renin-angiotensin system hyperactivation can induce inflammation and retinal neural dysfunction.”

*Michelle Callegan* *Michelle Callegan* *Meredith Gregory-Ksander* *Meredith Gregory-Ksander* *Mark Willcox* *Mark Willcox* *Susan Lightman* *Susan Lightman*

